# The experience of breastfeeding infants affected by congenital diaphragmatic hernia or esophageal atresia

**DOI:** 10.1186/s13052-018-0509-6

**Published:** 2018-07-03

**Authors:** G Salvatori, S Foligno, M Massoud, F Piersigilli, P Bagolan, A Dotta

**Affiliations:** Department of Medical and Surgical Neonatology, Bambino Gesù Children’s Hospital, Rome, Italy

**Keywords:** Breastfeeding, Breast milk (BM), Congenital diaphragmatic hernia (CDH), Esophageal atresia (EA), Human milk, Neonatal surgery unit (NSU), kangaroo mother care (KMC)

## Abstract

**Background:**

Newborns with congenital diaphragmatic hernia (CDH) and esophageal atresia (EA) might experience breastfeeding difficulties. The aim of this study was to detect the prevalence of breastfeeding in newborns with CDH and EA at different time points.

**Methods:**

We performed an epidemiological study and retrospective survey on the prevalence of breastfeeding in CDH and EA affected newborns. We identified 40 CDH and 25 EA newborns who were fed through breastfeeding procedures according to WHO categorized definitions, and compared the breastfeeding procedures at the beginning of hospitalization and at three months of life.

**Results:**

Although all the mothers attempted breastfeeding after birth, only 44 (67.7%) were still breastfeeding at the time of discharge. Exclusive breastfeeding was successful for only 19 (29%) mothers. The rate of exclusive breastfeeding at three months of life did not differ statistically from discharge and between the two groups of study.

**Conclusion:**

A large percentage of mothers of children with CDH and EA who breastfed at the beginning of hospitalization did not continue at three months. It would be important to increase the breastfeeding rate in CDH and EA affected newborns by following specific steps for vulnerable infants and sustaining breastfeeding after discharge.

## Background

Breast milk (BM) represents the optimum nutrition for full term newborns, sick neonates and preterm infants, because it contains anti-infectious properties, immune modulators, growth hormones and all the nutrients needed for physiological growth [1, 2]. Several studies have correlated exclusive breastfeeding with a reduced incidence of respiratory and gastrointestinal infections, nosocomial infections, re-hospitalization rates, and metabolic syndrome [3–5]. The advantages are particularly relevant in neonates undergoing surgery. BM reduces the incidence of complications in newborns undergoing surgery for cardiac abnormalities, malformations of the nervous system, or other organs [6–9]. BM is particularly advantageous in vulnerable infants because it is well tolerated and absorbed, allowing faster full enteral intake, early parenteral nutrition suspension, and earlier central venous catheter removal [10]. The first choice is to offer raw and unpasteurized mother’s milk; when the mother’s milk is not available, donor human milk is the recommended alternative [11, 12].

Newborns undergoing surgery for congenital diaphragmatic hernia (CDH) and esophageal atresia (EA) might particularly benefit from BM, because they are vulnerable infants. CDH is a developmental abnormality in which the diaphragm fails to form properly during embryogenesis, and consequently abdominal viscera invade the thoracic cavity, inhibiting pulmonary growth. Despite advances in treatment, mortality and morbidity rates in CDH infants remain high [13]. Morbidities linked to CDH include pulmonary disorders, cardiovascular and gastro-intestinal diseases, failure to thrive, neurocognitive defects, and musculoskeletal abnormalities [14, 15]. Gastro-esophageal reflux disease (GERD) is also an important contributor to overall morbidity, and can be responsible for feeding problems and failure to thrive [16, 17]. It is conceivable that an enteral feeding with a suitable choice like the human milk can improve the fasting tolerance and reduce GERD in CDH infants.

EA is a congenital interruption of the continuity of the esophagus with or without a persistent communication with the trachea. In 86% of cases there is a distal tracheo-oesophageal fistula, in 7% there is no fistulous connection, while in 4% there is a tracheo-oesophageal fistula without atresia [18]. Survival rates are reported to be around 90% in patients with, and almost 100% in term infants without associated anomalies [19]. Early and late complications occur more often in infants treated for a long gap atresia, and include tracheomalacia and GERD. GERD can be associated with long term feeding difficulties such as long mealtimes, food refusal, coughing, choking, vomiting, and delayed introduction of solids [20].

Our hospital is tertiary care referral centers for CDH and EA, and in our experience these condition represent two of the major cause of difficult to starting and continuation of breastfeeding. Therefore, we carried out a study of epidemiology and surveyed mothers to assess the prevalence of breastfeeding in newborns with CDH and EA at different time points.

## Methods

We performed a retrospective analysis of all infants who underwent surgery for CDH and EA from October 2011 through October 2014, and in January 2015 collected supplementary data after discharge by telephone interviews with the mothers of these previously hospitalized infants, at three months of life. The exclusion criteria were: need of surgical revision of EA, length of hospitalization over three months, and death before or immediately after surgical procedure. Most of the children had a prenatal diagnosis, and usually the birth of the child was planned. Children with CDH were electively intubated at birth and immediately transferred to our Neonatal Intensive Care Unit (NICU). When the vital parameters remained stable for 24 h, surgery was performed. Children with EA were not electively intubated, but they were also transferred immediately after birth to our NICU with a continuous suction in place, to reduce the incidence of pulmonary aspiration. Surgery was performed in the first days of life (first through fifth day of life).

Our Neonatal Intensive Unit (NICU) has dedicated staff to support breastfeeding: psychologists who support mothers through breastfeeding difficulties, and one International Board Certified Lactation Consultants (IBCLC) not present in the Neonatal Surgery Unit (NSU). Every year we organize a training course to improve staff knowledge about breastfeeding practices. We follow and sustain breastfeeding by the ten expanded steps of WHO [21].

To reduce bias linked with enrollment and disparity of outcome, we also registered the following data: age of the mother at birth, parity of the mother, neonatal clinical characteristics such as gender, weight and age at surgery, at first enteral feeding, at achievement of full enteral feeding, and at achievement of first oral feeding. Age at first enteral feeding was defined as the day of life on which either formula or human milk was first provided. Full enteral feeding was defined as an enteral feeding volume, either oral or gavage, of 150 ml/kg/day. First oral feeding was defined as the first day in which the infant had the entire feeding orally, without gavage. We recorded the different practices of breastfeeding (human milk, including the donor’s and the own mother’s milk), referring to the World Health Organization (WHO) definition: exclusive breastfeeding, predominant breastfeeding (breast milk and non-nutritional fluids), complementary/partial feeding (breast milk and artificial milk or other solid or semisolid nourishments) and bottle feeding [22, 23]. We found some mothers who attempted/ tried to breastfeeding, which means performed skin to skin practice, given breast to newborn, in order to stimulate sucking reflex under nursing advice and supervision. We analyzed each case at the time of discharge from the hospital and at 90 days of life. Survey questions administered to the mothers during phone interviews on the duration of breastfeeding and the type of feeding. The questionnaire is reported in Table 1. The same physician performed all the telephone interviews. We compared the rate of breastfeeding between the two groups of population in order to identify differences and predictable factors that may influence difficulties of breastfeeding.

**Table 1:** Questions asked to mothers during phone interviews

Have you ever breastfed your baby?	
If it is yes, how long did you breastfed your baby?	
Did you given your milk directly whit nipple or throughout bottle first and then nipple?	
Did you remember if your baby has never taken your milk throughout naso-gastric tube? If it yes, how long?	
How long baby has taken your milk from bottle?	
At what baby’s age did you start breastfeeding?	
At what baby’s age did you start bottle-feeding?	
At discharge have you given only your milk, or artificial milk, or both, or your milk whit others liquids (like water or chamomille)?	
At 1 months of life of baby have you given only your milk, or artificial milk, or both, or your milk whit others liquids (like water or chamomille)?	
At 3 months of life of baby have you given only your milk or artificial milk, or both, or your milk with others liquids (like water or chamomille)?	

### Statistical analysis

Statistical analysis was carried out by using the Statistical Package for Social Sciences (SPSS Inc., Chicago, Ilinois, USA), version 13.0. Significance was defined as *p* < 0.05. We performed a comparison between CDH and EA population. The categorical variables were compared using χ^2^ Person and Fisher tests. The ordinal variables were compared using t-test and non-parametric test (test U di Mann –Whitney). Data are presented as mean +/− standard deviation (SD).

## Results

During the study period we admitted 64 newborns with CDH (47.6% males) and 36 newborns with EA (62.1% males). We excluded 10 newborns who underwent revision for EA, 5 infants hospitalized over three months, and 20 neonates who died before or immediately after surgery for CDH. We therefore enrolled 65 newborns (53.8% males), 40 (61.5%) with CDH and 25 (38.5%) with EA. All mothers completed the telephone survey.

### Neonates with CDH

The main characteristics of the CDH children are presented in Tables 2 and 3. The mother’s age at birth ranged from 19 to 39 years, and 17 (42.5%) of them had had a previous pregnancy. Two mothers had pregnancy disorders (one hypertension, and one type 2 diabetes mellitus and hypertension). At time of discharge 13 (32.5%) infants were exclusively breastfed, 11 (27.5%) were taking complementary feeding, and 16 (40%) were taking artificial milk. At three months of life 12 infants (30%) were exclusively breastfed, 8 (20%) were having complementary feeding, and 20 (50%) were on artificial milk (Table 3). Enteral or oral feeding was started exclusively with human milk (either mother’s or donor’s milk) in 29 newborns (72.5%). Five neonates (12.5%) were breastfed from the start, 5 (12.5%) were fed with bottle, 7 (17.5%) were fed both ways, and in 23 neonates (57.5%) human milk was delivered through a naso-gastric tube. From questionnaire, we found that the principal cause of breastfeeding’s discontinuation at three months of life was the fear of lack of sufficient milk for the baby’s growth.

**Table 2:** Characteristics of CDH and EA population

GA < 37 weeks	1 (2.5%)	
Side of CDH	Left 39 (95%)	Right 2 (5%)
Type of CDH*	Type A: 7 (17.5%)	
	Type B: 26 (65%)	
	Type C: 4 (10%)	
	Type D: 1 (2,5%)	
	Type C/D: 2 (5%)	
Characteristics of EA population		
GA < 37 weeks	6 (24%)	
Type of EA**	Type II: 2 (8%)	
	Type III: 23 (92%)	

*The classification report the size of defect propose by Tsao K et al

**Classification of Gross type

**Table 3:** Continues variables of CDH and EA population

		Days Median +/− DS (min-max)	Weight (g.) Median +/− DS (min-max)
Surgery	CDH	3.48 +/− 1.9 (3–10)	3164+/− 497 (2170–4600)
	EA	3.9 +/− 1.6 (1–9)	2616+/− 475 (1820–3876)
Start of feeding	CDH	11.6 +/− 10.9 (4–58)	3132+/− 446 (2380–4362)
	EA	13.4 +/− 8.6 (5–49)	2758+/− 481 (1860–3712)
Full enteral feeding	CDH	31.49 +/− 28.4 (9–130)	3295+/− 515 (2500–4585)
	EA	21 +/− 9 (12–56)	2804+/− 494 (2060–3690)
Nipple	CDH	24.5 +/− 25.7 (7–107)	3053+/− 582 (2330–4190)
	EA	29.4 +/−20.8 (10–90)	2857+/− 496 (1970–3970)
Bottle	CDH	36 +/− 89.8 (4–360)	3806+/− 1051 (2690–6620)
	EA	39.3 +/−25.8 (11–90)	3191+/− 517 (2430–3800)
Length of breastfeeding	CDH	134.1 +/−98.6 (15–330)	
	EA	112.8 +/−86.8 (15–330)	

### Neonates with EA

The main characteristics of the EA children are presented in Tables 2 and 3. The mother’s age at birth ranged from 21 to 40 years, and only one (4%) had had a previous pregnancy. One mother had type 2 diabetes mellitus. At time of discharge 6 (24%) infants were exclusively breastfed, 9 (36%) were taking complementary feeding and 10 (40%) were taking artificial milk (Table 4). At three months of life 3 (12%) infants were exclusively breastfed, 7 (28%) were taking complementary feeding, and 15 (60%) were taking artificial milk (Table 4). Enteral or oral feeding was started exclusively with human milk (either mother’s milk or donor’s milk) in 15 newborns (60%). Five neonates (20%) were breastfed from the start, 12 (48%) were fed with bottle, 7 (19,4%) were fed both ways and in in 12 neonates (48%) human milk was delivered through a naso-gastric tube. In contrast to CDH population, the principal reported cause of discontinuation of breastfeeding at three months of life was GERD.

**Table 4:** Comparison of feeding in CDH and EA populations

CDH			EA	
Feeding characteristics	At discharge	At 3 months of life	At discharge	At 3 months of life
Exclusive breastfeeding	13 (32.5%)	12 (30%)	6 (24%)	3 (12%)
Partial breastfeeding	11(27.5%)	8 (20%)	9 (36%)	7 (28%)
Artificial milk	16 (40%)	20 (50%)	10 (40%)	15 (60%)

#### Comparison between children with CDH and EA

Independently from the type of surgery, 44 mothers (67.7%) tried to breastfeed at discharge. No significant difference was found between neonates with CDH and EA in terms of types of feeding (exclusive breastfeeding, partial breastfeeding and artificial feeding). No significant correlation was found between rate of feeding with human milk and number of previous pregnancies, pregnancy risk factors, or mother’s age. Among children with CDH, a significant difference was found between children breastfed during the first week of hospitalization versus those exclusively breastfed at discharge, and those exclusively breastfed at three months of life: 29 newborns were breastfed at hospitalization. But only 13 continued exclusive breastfeeding at discharge (*p* = 0.02), and only 12 at 3 months of life (*p* = 0.001). Among children with EA there was no significant difference between children breastfed during hospitalization versus those exclusively breastfed at discharge or at three months of life (Table 4). In both populations neither weight nor the day at which the babies reached full enteral feeding influenced the rate of breastfeeding. No other variable studied was correlated with the rate of breastfeeding at discharge or at three months of life.

## Discussion

Our results show that the rate of breast-feeding is low in CDH and EA newborns versus what it was in 2014 in the Lazio region, where our hospital is located (65.4% at discharge) (http://www.salute.gov.it/imgs/C_17_pubblicazioni_2256_allegato.pdf), but the rate of breastfeeding did not change significantly after hospital discharge.

BM is important for surgical infants, because it provides a barrier to potential illness, and could be beneficial during the newborn period and hospitalization. An epidemiologic study showed that human milk improves the outcome in newborns who undergo gastroschisis repair, with lower rate of positive blood cultures, lower percentage of days on inotropes, and shorter length of stay [24]. The relationship between human milk and CDH is not as extensively studied as for other vulnerable infants [25]. These infants have significant morbidities such as respiratory and gastrointestinal problems, that could benefit from receiving human milk [25]. Ideally, every mother should be encouraged to breastfeed within half an hour of birth. When infants are born with complications, lactation must be initiated using a breast pump, if possible within the first 6 h after birth. During the next days, many mothers of sick newborns can become discouraged because they cannot directly breastfeed their babies. In our Department, after surgery for CDH, neonates are intubated and given total parental nutrition for a time that depends on their clinical conditions. As soon as possible, after surgery the babies are extubated and transferred in the NSU. After surgery for EA, patients are often intubated and sedated for a period of time that depends on the distance between the proximal and distal esophagus. Postoperative elective paralysis is adopted to avoid tension on the esophageal anastomosis in long gaps. Enteral feeding via the trans-anastomotic tube is started around seven days, but before starting oral feeding we perform a routine contrast study. Parenteral nutrition is used in almost 50% of these patients. Once extubated, the babies are transferred to the NSU. For both conditions, breastfeeding or non-nutritive sucking at the breast should be initiated promptly once an infant has been extubated. Generally, healthy newborns learn to suck competently within a few days after birth. Feeding influences the mother’s sense of competence, and thus, mother–child relationship. Sucking and swallowing is a physiological process that the neonate develops in the presence of a normal oral motor function, functional esophagus and gastrointestinal system, independently of whether s/he is breast or bottle-fed. Because infants who undergo CDH surgery often cannot be breastfed for a long time, it might be important to implement the oral practice (stimulation of oral behavior) [26].In our experience to stimulate lactation and rise the mother’s milk supply, we often use the supplemental nursing system (SNS) filled with fresh pumped breast milk by infant suckling [27].

Despite the fact that CDH and EA have different treatment and peri-operative complications, we have evidenced that both have breastfeeding difficulties. Sixty-seven % of the mothers tried to breastfeed their baby after the surgery, but the percentage had declined at the time of discharge. Therefore despite the desire to breastfeed, some factors lead to frequent failure. One possible explanation is that these mothers are immediately separated from their child, delaying both mother-child relationship and the development of a correct rhythm of hunger and satiety, as well as the oral skills needed to competently suck [28]. Furthermore, these infants are subject to frequent oral procedures (aspirations, introduction of nasogastric tubes), possibly causing them to perceive as painful all sensations associated with oral functions. After discharge, the major limiting factor to breastfeeding was feeding difficulty related to GERD, as highlighted during the telephone survey. Indeed, complementary feeding was frequently a choice of the mothers because it allowed a change in the consistency of the milk. Indeed, in the EA population there was a significant decrease in the rate of breastfeeding both during hospitalization and after discharge. Accordingly, it has been highlighted that in this population, GERD and feeding difficulties lead to malnutrition [29, 30] To prepare a mother for breastfeeding at home, she should be afforded maximal opportunity to breastfeed her infant during hospitalization. The medical and nurse staff of the NICU should encourage mothers to spend as much time as possible at their infant’s bedside to learn their infant’s behaviors and feeding clues. For infants born with complex surgical anomalies, additional practice was proposed by Edwards and Spatz [31]. The Kangaroo mother care (KMC) should always be promoted, because the skin-skin contact might prolong and support breastfeeding [32]. Health caregivers should be educated about the benefits of offering as soon as possible to the neonate non-nutritive sucking, of reducing the time of enteral feeding and of transferring the baby as soon as possible to the NSU, giving the mother the possibility to restore a correct mother-child relationship [33]. Indeed, nurse participation and knowledge has been shown to improve the rate of breastfeeding in NICU [34].

Limitations of our study were a small population that reflects the low prevalence of above diseases and a lack of a healthy control group, due to the fact that our Department does not have a birth center. Despite these limitations, we present an important overview of the breastfeeding rate in a tertiary NICU specialized in two rare diseases (CDH and EA) during hospitalization and after discharge.

## Conclusions

The percentage of exclusive breastfeeding and complementary feeding for children operated for EA and CDH is low both during hospitalization and after hospital discharge. These rates remain unchanged after three months of life. Children with EA are breastfed at lower frequency compared to those with CDH. The main barriers to breastfeeding are probably related to the surgery and the perioperative course. Our study shows that it is necessary to improve breastfeeding rates in neonates undergoing surgery for CDH and EA already during hospitalization. To our knowledge, this goal should be reached by following the steps proposed by Spatz, not always practiced [31, 34, 35]. A further benefit could be reached by following also the expansion of “The Baby-Friendly Hospital Initiative, Ten Steps” set down by WHO for the NICUs [11, 12, 21], were the babies are hospitalized following surgery. Adequate prenatal counseling, a dedicated room for breast pumping, KMC, training of nurses and doctors and IBCLC supervision should all be considered hospital standards. We also believe that it would be helpful to support breastfeeding after discharge, either with clinic visits and through telephone calls.

## Abbreviations

BF: Breastfeeding
BM: Breast milk
CDH: Congenital Diaphragmatic Hernia
EA: Esophageal Atresia
GERD: Gastro-esophageal reflux disease
HM: Human Milk
IBCLC: International Board Certified Lactation Consultants
KMC: Kangaroo mother care
NSU: Neonatal Surgery Unit
NSU: Neonatal Surgical Unit
